# Alignment of Boron Nitride Nanofibers in Epoxy Composite Films for Thermal Conductivity and Dielectric Breakdown Strength Improvement

**DOI:** 10.3390/nano8040242

**Published:** 2018-04-15

**Authors:** Zhengdong Wang, Jingya Liu, Yonghong Cheng, Siyu Chen, Mengmeng Yang, Jialiang Huang, Hongkang Wang, Guanglei Wu, Hongjing Wu

**Affiliations:** 1 Center of Nanomaterials for Renewable Energy, State Key Laboratory of Electrical Insulation and Power Equipment, Xi'an Jiaotong University, Xi'an 710049, China; zhengdong.wang@stu.xjtu.edu.cn (Z.W.); ljy950417@stu.xjtu.edu.cn (J.L.); chensiyu0901@stu.xjtu.edu.cn (S.C.); belleyoung@stu.xjtu.edu.cn@qq.com (M.Y.); huangjialiang@stu.xjtu.edu.cn (J.H.); hongkang.wang@mail.xjtu.edu.cn (H.W.); wuguanglei@mail.xjtu.edu.cn (G.W.); 2 Institute of Materials for Energy and Environment, Growing Base for State Key Laboratory, College of Materials Science and Engineering, Qingdao University, Qingdao 266071, China; 3 Department of Applied Physics, Northwestern Polytechnical University, Xi’an, 710072, China; wuhongjing@mail.nwpu.edu.cn

**Keywords:** electrospinning technique, BN nanofibers, alignment, epoxy composite, thermal and dielectric properties

## Abstract

Development of polymer-based composites with simultaneously high thermal conductivity and breakdown strength has attracted considerable attention owing to their important applications in both electronic and electric industries. In this work, boron nitride (BN) nanofibers (BNNF) are successfully prepared as fillers, which are used for epoxy composites. In addition, the BNNF in epoxy composites are aligned by using a film casting method. The composites show enhanced thermal conductivity and dielectric breakdown strength. For instance, after doping with BNNF of 2 wt%, the thermal conductivity of composites increased by 36.4% in comparison with that of the epoxy matrix. Meanwhile, the breakdown strength of the composite with 1 wt% BNNF is 122.9 kV/mm, which increased by 6.8% more than that of neat epoxy (115.1 kV/mm). Moreover, the composites have maintained a low dielectric constant and alternating current conductivity among the range of full frequency, and show a higher thermal decomposition temperature and glass-transition temperature. The composites with aligning BNNF have wide application prospects in electronic packaging material and printed circuit boards.

## 1. Introduction

In recent years, to achieve a better performance, the tendency in electronic technology has been towards integrating more transistors into a single chip. Yet this has caused the escalation of energy dissipation and significant heat fluxes in a device, which negatively influences its lifetime and reliability [1,2,3,4,5]. So, novel materials with better electrical and thermal performance are needed. Epoxy resin is one of the most frequently used insulation materials, with superior electrical and machining properties [6,7]. However, its low thermal conductivity greatly restricts its applications because of the increasing heat produced by unit volume of equipment [8,9]. To improve thermal conductivity of epoxy resin, the fillers of micro-scale with high thermal conductivity can be considered. Nevertheless, the dilemma for using micro-sized inorganic particles is that the increase of thermal conductivity is usually attained at the expense of a significant decrease in breakdown strength, which thus limits their applications in high output electrical devices [2,10,11]. Thus, it can be seen that the development of epoxy-based composites with high thermal conductivity and dielectric breakdown strength is impending.

In the field of electrical insulation, inorganic particles with high intrinsic thermal conductivity and desirable insulating properties are considered more as fillers of composites, such as alumina (Al_2_O_3_), aluminum nitride (AlN) and boron nitride (BN) [2,8,12,13,14]. Among these fillers, boron nitride has become a research focus because of its high thermal conductivity (intrinsic ~2000 W/m K), low permittivity ε’ ~ 4, loss and density. In addition, for the high aspect ratio, advances are focused on one-dimensional boron nitride nanotubes (BNNTs) and two-dimensional boron nitride nanosheets (BNNSs) [1,15,16].

For 2D thermal transport materials, graphene polymer composites exhibit high thermal transport and high electric conductivity, exhibiting many applications in the field of electronics [17,18,19]. BNNSs are very promising materials for application in the electrical insulating field. But there are still some problems and bottlenecks that need to be addressed. Different from graphene, BNNSs are brittle and have a stronger interlayer interaction, making them difficult to exfoliate. For 1D materials, similar to carbon nanotubes (CNTs), boron nitride nanotubes (BNNTs) have a single-walled or multi-walled tubular hexagonal structure [20,21,22]. It is worth noting that BNNTs have a large band gap and superior thermal transport properties while CNTs are conductive. Hence, BNNTs have great potential for high thermal conductivity and insulating composite materials [1,23]. However, the preparation methods for BNNT, such as the arc-melting method and Chemical Vapor Deposition, are complex to operate [24,25,26]. Moreover, the yield of BNNTs is low and the control of the perfect structure is fairly difficult, limiting its practical application for significantly improving thermal conductivity.

In this work, the electrospinning method is applied to prepare the nanofibers. Recently, the electrospinning method has attracted more and more attention due to its many advantages, such as easy preparation technology, high-yield nanomaterials and regular structure of nanofibers for energy storage and thermal management materials [27,28,29,30]. In addition, nanofibers prepared by electrospinning have a large aspect ratio because of their long length and controllable diameter, which is beneficial for forming a connected network for the improvement of thermal conductivity of nanocomposites [31,32,33]. Moreover, dielectric fillers with a high aspect ratio are found to be effective in improving breakdown strength [23]. Furthermore, it was found to be possible that breakdown strength may be improved as the nanofibers oriented preferentially in the in-plane directions of the composite films [34]. In order to realize the orientation of the nanofibers, the coating method would be applied. In this paper, the orientation-BN nanofiber/Epoxy composite film with increasing breakdown strength and thermal conductivity is developed. Utilizing the electrospinning method to fabricate the BN nanofibers and applying the coating method to orient the nanofibers inside the composite film. The electrical performance of the products will be evaluated including dielectric properties and the electrical breakdown strength at a power frequency alternating current (AC) system; On the other hand, BNNF incorporated into polymer can enhance the thermal-mechanical dielectric properties of polymer composites by restraining the free mobility of polymer molecular chains, such as thermal stability and glass-transition temperature, which are also crucial for packaging materials in powerful micro-electronic devices [35,36,37,38,39]. The thermal performance of the products has been evaluated, including thermal conductivity, thermal gravimetric analysis (TGA) and differential scanning calorimetry (DSC).

## 2. Experimental

### 2.1. Material

Polyvinyl butyral (PVB) powder was bought from Sinopharm Chemical Reagent (Shanghai) Co. Ltd., China. Boric oxide (B_2_O_3_) was purchased from Aladdin Reagent (Shanghai) Co. Ltd., China. Absolute ethyl alcohol was purchased from Zhiyuan Chemical Reagent (Tianjin) Co. Ltd., China. The bisphenol A epoxy resin (E-828) along with anhydrite hardener (MTHPA) and benzyldimethylamine (BDMA, used as the accelerant) were purchased from Aladdin Reagent (Shanghai) Co. Ltd., China.

### 2.2. Preparation of BNNF

**Figure 1:**
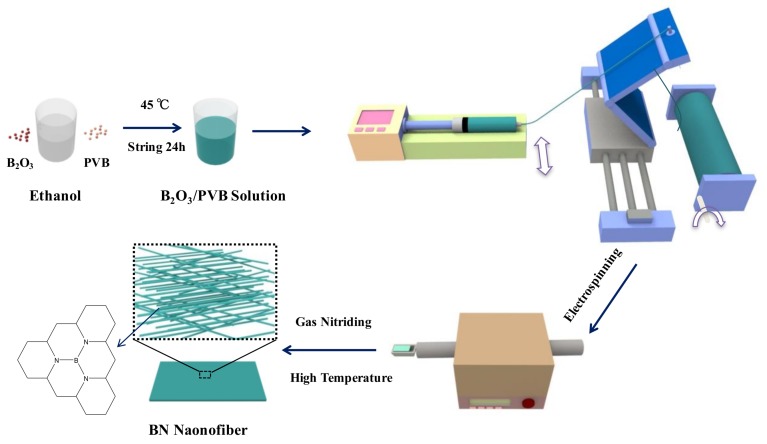
Schematic illustration showing the fabrication of boron nitride (BN) fiber via electrospinning.

As shown in Figure 1, first, 1.4 g of boron oxide powder was dissolved in 20 mL of absolute ethanol solvent and stirred at 45°C with a magnetic stirrer until completely melted. Then, 0.7 g of PVB (M_w_ = 60,000) powder was dissolved in 20 mL of absolute ethanol solvent and poured into B_2_O_3_/ethanol solvent. The mixture was stirred vigorously at 45 °C for 24 h. Next, the electrospinning method was used to prepare BN nanofibers. The output voltage of electrospinning was 16 kV. Besides, to prevent occluding the needle with precipitate from solvent evaporation during the process of electrospinning, the syringe propeller was applied to control the flow rate of the jet flow. The prepared solution was injected via a syringe with a feeding rate of 1.5 mL/h. Then, the PVB is removed from the B_2_O_3_/PVB fiber (BOF) and the BOF was nitrided. The BOF was put into a crucible, which was large enough to make the fiber stretch; the crucible was put into a sintering furnace with a 10% O_2_/90% NH_3_ (vol/vol) gaseous mixture with a total flux of 200 mL/min, while at the same time, heating it from room temperature to 800 °C. After 2 h of reaction under 800 °C, the polymer from the BOF was removed. The gaseous mixture was converted into NH_3_ (99.99%) at 100 mL/min, heating from 800 °C to 1100 °C and then held at 1100 °C for nitriding for 6 h. Secondly, the atmosphere changed into N_2_ (99.99%) at 200 mL/min, heating from 1100 °C to 1500 °C with a rate of 1 K/min and was then held at 1500 °C for nitriding for 4 h. Finally, the boron nitride nanofibers (BNNF) were obtained and preserved for the preparation of nanocomposites.

### 2.3. Preparation of Epoxy-based Nanocomposites Film

According to a proportion of epoxy/hardener/accelerant of 1/0.86/0.02, the weight of BNNF we needed was calculated (0.5 wt%, 1 wt% and 2 wt%). First, epoxy was poured into a beaker and added to the BNNF, and was subjected to high-speed mechanical stirring until the nanofibers were dispersed well; then the hardener and the accelerant were poured in and stirred at a high speed. The mixture was put into the vacuum oven for removing bubbles at 60 °C for 20 min. 

Before the automatic coating machine was used, the machine should lay a layer of polyethylene film and be preheated at 100 °C—the thickness of the coating was 100 μm. Next, the air pump was turned on, which made the polyethylene film attach tightly to the machine. The moderate BNNF/epoxy suspension was wiped by the automatic coating machine and poured into a hollow model with a thickness of 100 micron, a polyethylene film was attached to the suspension to prevent the epoxy nanocomposites shrinking. The film was heated at 100 °C until the suspension was completely dried. After that, the film was transferred into the oven for curing. The procuring was held at 100 °C for 2 h; the post curing was held at 150 °C for 10 h. Finally, when the temperature cooled down to room temperature, the EP/BNNF film was taken out with the polyethylene film and the EP/BNNF film was carefully stripped out.

### 2.4. Characterization

The morphological structures of the fillers and the surface morphology and fractured morphology of the composites were characterized using field emission scanning electron microscopy (FESEM, FEI QUANTA F250, Hillsboro, TX, USA). The microstructure of the BNNF and EP/BNNF film was observed with an FEI Tecnai G2 F20 S-TWIN (Hillsboro, TX, USA) transmission electron microscope (TEM). The phase structure of the prepared BNNF was tested with X-ray diffraction (XRD) using a Bruker D2 PHASER diffractometer (Karlsruhe, Germany). The thermal diffusivity (α) and specific heat (C_p_) were measured with an LFA 467 Nanoflash (NETZSCH, Selb, Germany). The bulk density (ρ) of the sample was given by the product of the length, width and height. The thermal conductivity value of the samples was calculated by the product of the thermal diffusivity, specific heat, and bulk density. The equation is λ = αρC_p_. In the test of characterization, the samples were cut into a quadrate shape, 10 × 10 mm^2^ in proportion and 0.3 mm in thickness. For the dielectric property measurements, gold electrodes were first sputtered on both sides of the epoxy-based composites by the auto fine coater with a mask that had 3 mm diameter eyelets. The dielectric properties of the composites were measured with a broadband dielectric spectrometer (CONCEPE 80, Novocontrol Technology Company, Montabaur, Germany) with an Alpha-A high-performance frequency analyzer over the frequency range of 10^−1^ Hz to 10^6^ Hz. Thermal gravimetric analysis (TGA) was performed with a heating rate of 60 °C/min under nitrogen. Differential scanning calorimetry (DSC, 200 F3, NETZSCH, Selb, Germany) was performed at temperatures from 25 °C to 200 °C at a heating rate of 10 °C/min under a nitrogen atmosphere to study the glass transition temperature (T_g_) of the composites. The electrical breakdown strength at the commercial power frequency of composites was measured using the standard of IEC 60243.

## 3. Results and Discussion

### 3.1. X-ray Diffraction, SEM and TEM Analysis (BNNF)

**Figure 2:**
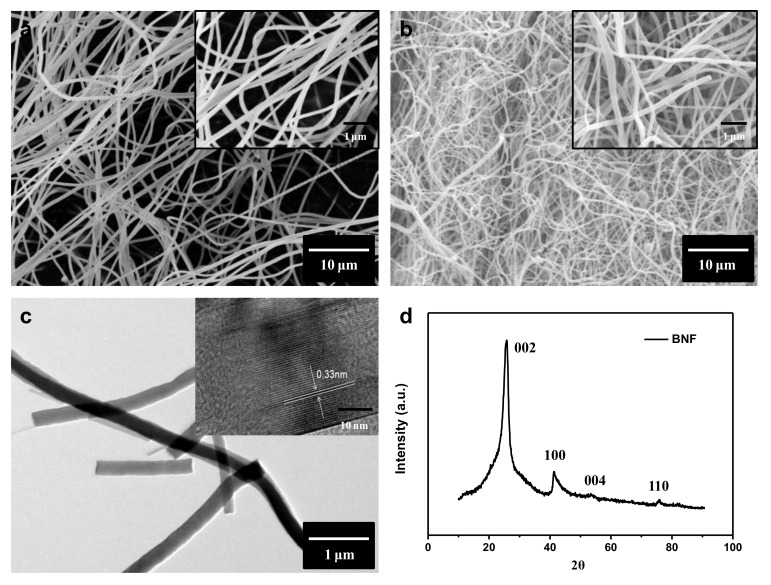
Scanning electron microscopy (SEM) image of (**a**) B_2_O_3_/PVB composite fiber and (**b**) BN fiber samples with insets showing the corresponding enlarged views. (**c**) Transmission electron microscopy (TEM) images of BN fiber with HRTEM image in the inset. (**d**) X-ray diffraction (XRD) pattern of BN fiber.

We utilized the scanning electron microscope (SEM), transmission electron microscope (TEM) and X-ray diffraction (XRD) to characterize the morphology and elements of PVB/B_2_O_3_ composite fiber and the prepared boron nitride nanofiber. As shown in Figure 2a, PVB/B_2_O_3_ composite fiber is thin and has a smooth morphology with a uniform diameter ranging from 150 nm to 200 nm, when the flow rate is 1 mL/h. Figure S1a–f shows the morphology of PVB/B_2_O_3_ fiber prepared with a different flow rate. According to these SEM photos, it was found that in the circumstance of the same solution proportion and voltage, when the flow rate is more than 1 mL/h, the surface of the fiber becomes coarser and forms a structure of beads and the diameter of the fiber tends to be bigger with increasing flow rate. Figure 2b shows the SEM photograph of BNNF (inset is an enlarged view). It can be seen that the surface of the BN fiber is smooth and the average diameter of a BN fiber is around 150 nm (see Figure S2). Figure 2c shows TEM images of BN nanofibers with an high resolution transmission electron microscope (HRTEM) image in the inset. It can be seen that the lattice spacing is 0.33 mm in its high-resolution image, which is the typical value of BN lattice spacing [40]. The XRD pattern of BNNF is shown in Figure 2d. BN fibers exhibited a well-crystallized hexagonal structure. The peaks at approximately 26.9, 41.6, and 54.9 are assigned to the (002), (100), (004) and (110) crystallographic planes of h-BN, respectively [41]. These peaks are typical of h-BN according to the MDI Jade database. There were no other impurity phases being detected, also indicating the high purity of BN nanofibers. The high crystalline quality of BN fillers was beneficial to improving the thermal conductive and electrical properties of the composites.

### 3.2. SEM and Optical Microscope Analysis of BNNF/Epoxy Composites

**Figure 3:**
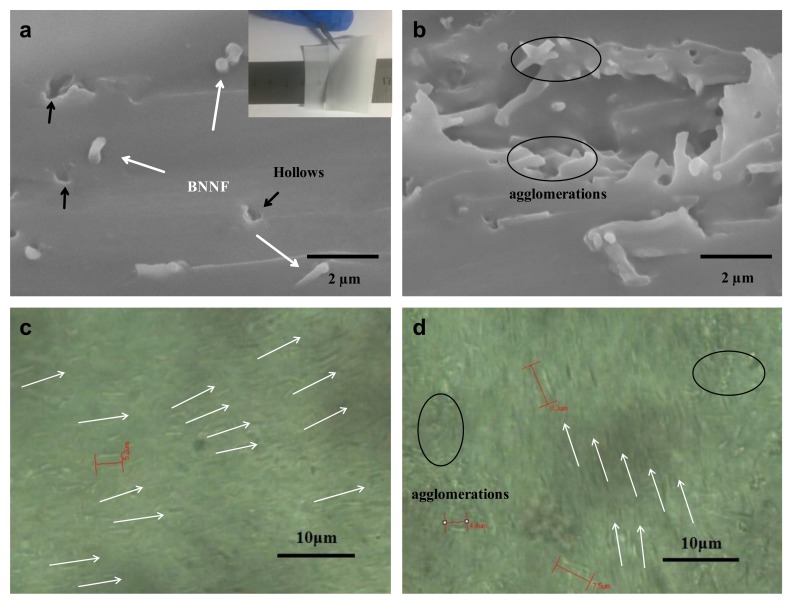
SEM cross-section image of (**a**) 1 wt% BNNF/EP and (**b**) 2 wt% BNNF/EP film. The inset in (**a**) shows the flexibility and transparency of sample. Optical microscope surface photograph of (**c**) 1 wt% BNNF/EP and (**d**) 2 wt% BNNF/EP composite film.

Figure 3a,b show the cross images of BNNF/EP film with 1 wt% BNNF and 2 wt% BNNF, respectively. According to Figure 3a, the thread ends of BNNF can be seen and there are some hollows (black arrows) dispersed on the cross section, which were caused by fractured BN nanofibers in liquid nitrogen, while BNNF nanofibers were uniformly dispersed in the epoxy matrix. Recently, the alignment of 1D and 2D nanomaterials has been widely applied to prepare anisotropic functional materials. For instance, boron nitride nanosheets and graphene polymer composites can dramatically increase thermal conductivity in the in-plane direction because of their anisotropic performance. The A.A. Balandin research group aligned the graphene functionalized with Fe_3_O_4_ nanoparticles by applying an external magnetic field. The thermal conductivity of composites with low loading oriented graphene reaches up to 1.3 W/m⋅k [42]. G. Lazzara et al. developed Halloysite as a tubular template to obtain ordered arrays of clay nanotubes on solid substrates with multifunction including controlled release, good mechanical properties and anti-water [43,44,45,46]. Indeed, many methods such as filtration, freezing ice, hot pressing, shear or extrusion flow, stretching, electrospinning, electric field, magnetic field and so forth, have used to acquire the oriented nanofillers in polymer matrix. In this work, the casting method is applied to align the BNNF in the epoxy matrix via drawknife. The drawknife provided the propelling force on the side of nanofibers, and compelling the fiber to lie down in the epoxy matrix. In the other words, the lying fibers in the polymer were more stable than the standing fibers when they were loaded the planus force, perpendicular to the standing direction. Most thread ends of BNNF in epoxy are perpendicular to the cross section of the composite, which demonstrated the good orientation of BNNF. The reason is that the drawknife provided an external force on the BN fiber in composites during the coating of the suspension with epoxy and BNNF, resulting in the formation of a BNNF carpet [34]. In other words, the drawknife produced a push force on BNNF, leading to the fall of BNNF perpendicular to the drawknife. Moreover, the clear fiber-like structure (BN fiber) was observed by optical microscope and their length was within the region of 4–9 μm. Optical microscope images of BNNF/epoxy composites (Figure 3c) are more macroscopic to show the uniform dispersion and alignment of BNNF (white arrows showing the aligned orientation) in the epoxy matrix. To exhibit the good flexibility and transparency properties of our BNNF-epoxy film, we folded the film (inset in Figure 3a) with tweezers over the ruler, which demonstrated the good mechanical properties and homogeneous dispersion of BNNF in composites. As shown in Figure 3b, some BN nanofibers formed agglomerations in composites due to the high content and surface area and energy of BNNF. Correspondingly, the partial agglomerations of BNNF in epoxy composites with 2 wt% BNNF can also be found in the optical microscope images of the BNNF/epoxy composites (Figure 3d). Despite all this, we can still find the obvious alignment of BNNF (as indicated by the white arrows) in epoxy composites. 

### 3.3. Dielectric and Thermal Properties

**Figure 4:**
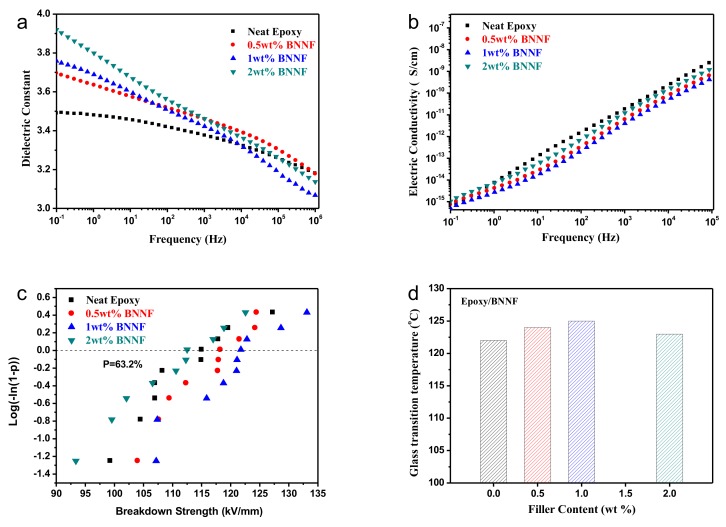
(**a**) Dielectric permittivity(ε’) and (**b**) electric conductivity dependence on frequency and filler loading for the epoxy composite film with different mass fraction of BNNF at room temperature. (**c**) Weibull plots of breakdown strength of Epoxy/BNNF composite film. (**d**) DSC curves of four samples of different mass fraction of BNNF.

As expected, the dielectric permittivity increased with the increment of the filler loading in epoxy over all frequency ranges from 10^−1^–10^6^ Hz at room temperature (298 K) for the epoxy composites with different BNNF mass fractions. Figure 4a,b show the relationship between permittivity(ε’) and dielectric loss(tanδ) of BNNF/epoxy composites film with various BNNF mass fraction and frequency. In BNNF/epoxy composite systems, mainly 3 parts of dielectric loss are included as follows: (1) the conduction loss caused by incorporated inorganic fillers; (2) the relaxation loss gave rise to the polar functional groups of polymer matrix; (3) interface loss from the interface between filler and polymer matrix. According to Figure 4a, as the frequency increased, the permittivity of all the samples tended to decrease. It can be attributed to the lower influence of relaxational polarization, which was unable to keep pace with the high frequency change so that displacement polarization became dominant. Therefore, the permittivity of composites decreased. The permittivity of pure epoxy film was around 3.5 under the frequency of 1 kHz and the permittivity of most BNNF/EP composite samples were slightly higher. This would be explained by the interfaces among fillers and epoxy resin interacting to cause interfacial polarization, which is a significant factor for the increase of permittivity of composite material. As shown in Figure 4a, in low-frequency regions, the value of permittivity of composite increased as increasing the amount of BNNF. This could be interpreted as the larger the content of BNNF, the comparatively larger the interface boundary between filler and matrix; thus, the interfacial effect and polarization became stronger so that the permittivity became slightly higher. However, in high-frequency regions, the relaxation polarization in the composite could not follow the change of frequency [7]. As the influence of interface polarization on the permittivity was less, the value of permittivity of all the samples decreased and tended to continually decrease with increasing frequency. What is more, the dielectric constant of the composite with 1 wt% BNNF is lower than that of other composites in high frequency zones. The high-frequency dielectric response can be accounted for by the C-F dipole orientation polarization of the composites based on the Debye relaxation theory [13]. The dielectric properties of polymer incorporated by semiconductors or insulators have close contact with the electrical properties of inorganic fillers, significantly affecting the accumulation and migration of free carriers at the interaction area between the filler and the polymer matrix. The dielectric constant of BNNF/epoxy composites is lower than that of pure epoxy.

Figure 4b presents the dependence of electric conductivity on frequency at room temperature of BNNF/epoxy composites. The electric conductivity of all samples increases with the increment of the tested frequency. The dielectric constant of the composites with BNNF is lower than that of neat epoxy at all tested frequency ranges from 10^−1^ to 10^6^ Hz. The reasons can be ascribed to the high intrinsic resistance of BNNF and the interface layer between polymer and fillers according to the core-shell theory [9], which blocks the direct transfer of free charge carriers and prolongs the transport path of carriers in the epoxy matrix. Therefore, one can declare that the incorporating BNNF plays a crucial role in suppressing the conduction of the epoxy composites. 

The breakdown field strength (BD) and its Weibull distribution of the breakdown test of epoxy nanocomposites with 0.5 wt%, 1 wt%, 2 wt% BNNF and pure epoxy are shown in Figure 4c. According to the experimental results in Figure 4c, the BD strength of the epoxy composites with 0.5 wt% BNNF was slightly higher than that of pure epoxy film; the BD strength of the sample of 1 wt% BNNF was higher obviously; but the BD strength of the sample of 2 wt% BNNF was slightly lower than that of the pure epoxy film. In general, that doping micro-sized inorganic fillers into polymer would decrease the BD strength of the polymer itself [2,11]. First, surface energy of micro-sized filler is weak and polymer molecule cannot closely attach to micro filler, leading to the obvious incompatible interface between filler and polymer matrix; Second, dielectric permittivity of inorganic filler is much higher than that of polymer which would cause large-scale electric field distortion in the polymer composite, leading to the decrease of BD strength; The other reason is that the voids and impurity caused by the doping fillers make the BD strength of composite lower than that of pure polymer. However, in this work, specific surface area of BNNF is large. Actually, the large surface area of nanomaterials has many applications such as battery, wave absorption and electromagnetic properties and so forth [47,48,49,50,51,52,53,54,55,56]. In other words, BNNF has high surface energy, which is beneficial to form closer interface. In addition, it is worth noting that BNNF has lower dielectric constant in comparison with other high thermal conductive inorganic filler (such as AlN, Al_2_O_3_ and SiC). Dielectric constant of BNNF approaches to that of pure epoxy, and the close permittivity between BNNF and epoxy relieves field distortion of BNNF/epoxy composites. Moreover, BNNF prepared by our method is very purified and has no other impurity phases, preventing the effect on BD from impurity phases. In addition, the number and size of voids and bubbles are effectively reduced by using hot-pressing technology. From our results, the moderate amount of doping BNNF in epoxy resin can increase the BD strength of composite. It can be explained that the doping BNNF is directionally distributed near the interface of the film and the oriented nanofibers of tubule are perpendicular to the electric field direction so that will signally scatter electrons, which could effectively restrain the growth of electrical tree and extend the growth path of electrical tree thus put off the forming of penetrating breakdown. Therefore, the BD strength of the composite increases. Nevertheless, as the mass fraction of BNNF continually increases, the BD strength of composites decreases. It is attributed to the more amount of fillers, the more aggregation of nanofibers in the composites and the more difficulty of fillers distribution in matrix, resulting in decrease in the BD strength of the samples.

The DSC curves of pure epoxy, 0.5 wt%, 1 wt% and 2 wt% BNNF film are shown in Figure 4d. The glass transition temperature (T_g_) of the pure epoxy film is around 121 °C Compared with that of epoxy, the T_g_ of epoxy composites with 0.5 wt% and 1wt% BNNF is 125 °C and 126 °C, respectively (See Figure S2). However, the Tg of epoxy composite with 2 wt% BNNF shows slightly decreased in comparison with that of composites with lower BNNF mass fraction. It can be attributed to the uniform dispersion of BNNF in epoxy matrix (see Figure 3a,c), resulting the more interface between filler and epoxy. In addition, a reason for this observation is attributed to the interfacial interaction between BN fiber fillers embedded in composites and epoxy matrix, which restricts the movement of the polymer molecular chain and restrains the thermal decomposition, leading to the higher T_g_ and increasing the thermal stability of epoxy matrix (see Figure S3). The BNNF has decent thermal conductivity and thermal stability properties, which can effectively transfer heat. Meanwhile, the measuring *C_p_* data and their accuracy are summarized in Table S1 (See the Supporting information). The Cp of pure epoxy is higher than that of BNNF/epoxy composites. It is attributed to the good mobility of neat polymer. BNNF incorporated into polymer retained the free mobility of the polymer, resulting in lower *C_p_*. Moreover, the intrinsic *C_p_* of BNNF is lower in comparison with that of epoxy matrice.

### 3.4. Thermal Conductivity

The as-fabricated BNNF/epoxy nanocomposite films were evaluated for their thermal transport properties by using a commercially acquired instrument based on the Laser flash method [37,38], measuring the thermal diffusivities (TD) and specific heat (*C_p_*) in each film. It is worth noting that thin gold electrodes (around 10 nm) need to be sputtered on both sides of films by the auto coater to delay laser transmitting time. In addition, a spot of graphite was sprinkled on the surface of gold coating for receiving the heat from laser. The accuracy of the instrument for specimen of TD and *C_p_* was evaluated and validated by measuring and comparing the standard glass (Pyrex7740), from which the average deviations with respect to the use of different sets of instrumental parameters and in repeated measurements were generally less than 10%. For the BNNF/epoxy nanocomposite films, multiple measurements of several specimens yielded TD values shown in Table 1 and corresponding *C_p_* and density data were shown in Table S1. When the temperature is constant at T, the calculation relationship between thermal conductivity and TD is as follows [5]:

(1) $$\lambda \left(T\right)=\alpha \left(T\right)\cdot C_{p}\left(T\right)\cdot \rho \left(T\right)$$

where *λ* is thermal conductivity, α is thermal diffusivity, *C_p_* is specific heat and *ρ* is bulk density of sample.

**Figure 5:**
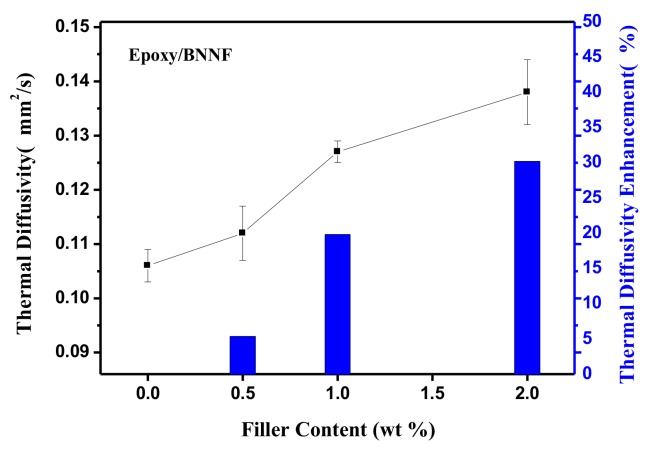
The thermal diffusivity and enhancement of four samples with different mass fraction of BNNF.

As expected, the thermal diffusivities of the polymeric nanocomposites are strongly dependent on the BNNF content, with higher mass fractions consistently resulting in higher thermal diffusivities of the nanocomposites. Figure 5 shows the dependence of the thermal diffusivity on the mass fraction of nanofiber in the BNNF/epoxy nanocomposite films. From the Figure 5, as the mass fraction of BNNF increased, the thermal diffusivity of the composites increased obviously. Meanwhile, thermal conductivity of nanocomposites increased with increment of BNNF. The thermal conductivity value of composite with 2 wt% BNNF increased to 0.205 W/mK (shown in Table 1 and their accuracy shown in Table S1), which enhanced 26% than that of pure epoxy. It is well known that the heat conduction mechanism of BN is phonon transfer which caused by lattice vibration. As the mass fraction of filler increased, the quantity of microcosmic interface between filler and epoxy-base increased, which increased the chance of phonon scattering that restrained the transfer of phonon. Nevertheless, when the fillers were added to a certain amount that long nanofibers were easier to connect to each other, it would form a partial thermal transport network. 

Nielsen proposed a relatively simple model for the evaluation of thermal conductivities of a composite material [57]. In his approach, the thermal conductivity of a composite material, *Kc*, is related to the thermal conductivity of a matrix, *K_m_*, and a filler, *K_f_*, according to the following equation:

(2) $$K_{c}=K_{m}[\left(1+ABV_{f}\right)/\left|1-B\beta V_{f}\right|$$

where the parameters *B* and β are given by

(3) $$B=[\left(K_{f}/K_{m}-1\right)/\left(K_{f}/K_{m}+A\right)$$

(4) $$\beta =\left[\left(1-V_{m}\right)/V_{m}^{2}\right]V_{f}+1$$

here, *V_f_* represents the volume fraction of the filler, A is a constant related to the generalized Einstein coefficient reported for most of the materials, [57] and *V_m_* is the maximum packing fraction. 

The experimental thermal conductivity values for BNNF/epoxy composites are in agreement with the calculated results by the Nielsen’s model (see Table 1). It is worth noting that Terao et al. have reported on the BNNTs/PVA composites [58]. In that report, The BN nanotubes were used as the fillers and the thermal conductivity behavior of composites incorporating oriented and random fillers was researched by using the Nielsen’s model. In their research, the theoretical results were significant different with experimental results. The theoretical results showed that thermal conductivity sharply increased when the BNNTs content in a composite with randomly dispersed BNNTs was more than 30 vol%. However, the similar increase took place when the BNNTs fraction exceeded 10 vol% (for the aligned BNNT case). In other words, the percolating value for the increase in thermal conductivity decreased to 10 vol% when BNNTs were oriented in composites. Therefore, alignment of one-dimensional fillers such as BNNTs and CNTs can enhance thermal conductivity of composites under the condition of containing same fillers volume fraction [58,59]. It can be attributed to closer distance between fillers and the decrease of percolating fraction in polymer. Moreover, the low volume fraction of fillers incorporated in polymers can enhance the mechanical and electrical properties of polymers in comparison with the high content filler in composites. The theory indicates that the thermal conductivities of BNNF/epoxy composites have still big room for improvement of if one manages to incorporate higher fractions of BNNF into a polymer while maintaining good alignment of the fillers. 

## 4. Conclusions

In summary, the addition of BNNF to the epoxy composites has no obvious effect on the dielectric permittivity of epoxy. However, the electric conductivity of the composites was lower than that of pure epoxy within the tested frequency region due to the high intrinsic resistance of BNNF and the interface layer between polymer and fillers, which blocks the direct transfer of free charge carriers and prolong the transport path of carriers in epoxy matrix. Adding moderate mass fraction of BNNF improved the breakdown strength of the composites: the breakdown strength of epoxy composite film with 1 wt% BNNF was 122.9 kV/mm, increased 4.8% compared with that of pure epoxy film (117.2 kV/mm); the addition of BNNF to the composites also influenced its thermal properties. The interfacial effect was formed after doping the fillers into the epoxy resin, which improved the heat transmission temperature and the thermal stability of the composites. Furthermore, due to the superior heat-conducting property of BN itself, the thermal conductivity values of the BNNF/epoxy composite films were enhanced. The thermal conductivity of 2 wt% BNNF/epoxy film was increased 26.4% compared with the pure epoxy film.

## Figures and Tables

**Table 1:** Thermal diffusivity and dielectric breakdown strength of samples with different mass fraction of BNNF.

Samples	λ (W/m⋅k)	Nielsen (W/m⋅k)	α (cm^2^/s)	DBS (kV/mm)	β	Thickness (μm)	Deviation (μm)
Pure Epoxy	0.162	0.162	0.106	115.8	14.74	112	±8
0.5 wt%	0.17	0.181	0.112	118.8	20.95	108	±3
1 wt%	0.185	0.194	0.127	122.8	19.67	112	±7
2 wt%	0.205	0.227	0.138	113.5	15.05	115	±3

λ: thermal conductivity α: thermal diffusion coefficient, DBS: Dielectric Breakdown Strength, β: Shape parameter.

## Supplementary Materials

The following are available online at http://www.mdpi.com/2079-4991/8/4/242/s1, Figure S1: The Proportion of PVB/B_2_O_3_ is 700 mg/350 mg, the voltage is 16.14 kV, the flow is (a) 0.75 mL/h (b) 1.5 mL/h (c) 2.0 mL/h (d) 2.5 mL/h (e) 4.0 mL/h (f) 1.0 mL/h. Figure S2: DSC curves of the BNNF/epoxy composites with different filler content under nitrogen atmosphere. Figure S3: TGA curves of the BNNF/epoxy composites with different filler content under nitrogen atmosphere. Table S1: A summary of the detailed data information on thermal conductivity.
