# Supplementary material for: Alignment of Boron Nitride Nanofibers in Epoxy Composite Films for Thermal Conductivity and Dielectric Breakdown Strength Improvement
Source: Nanomaterials (Basel). 2018 Apr 15;8(4):242. doi: 10.3390/nano8040242 (PMC5923572; doi:10.3390/nano8040242)
Supplement: Supplementary file 1 [file nanomaterials-08-00242-s001.pdf]

Supplementary Materials

Alignment of Boron Nitride Nanofibers in Epoxy Composite Films for Thermal Conductivity and Dielectric Breakdown Strength Improvement

Zhengdong Wang [1,†], Jingya Liu [1,†], Yonghong Cheng [1,*], Siyu Chen [1], Mengmeng Yang [1], Jialiang Huang [1], Hongkang Wang [1], Guanglei Wu [1,2] and Hongjing Wu [3]

[1] Center of Nanomaterials for Renewable Energy, State Key Laboratory of Electrical Insulation and Power Equipment, Xi'an Jiaotong University, Xi'an 710049, China; zhengdong.wang@stu.xjtu.edu.cn (Z.W.); ljy950417@stu.xjtu.edu.cn (J.L.); chensiyu0901@stu.xjtu.edu.cn (S.C.); belleyoung@stu.xjtu.edu.cn@qq.com (M.Y.); huangjialiang@stu.xjtu.edu.cn (J.H.); hongkang.wang@mail.xjtu.edu.cn (H.W.); wuguanglei@mail.xjtu.edu.cn (G.W.)

[2] Institute of Materials for Energy and Environment, Growing Base for State Key Laboratory, College of Materials Science and Engineering, Qingdao University, Qingdao 266071, China

[3] Department of Applied Physics, Northwestern Polytechnical University, Xi’an, 710072, China; wuhongjing@mail.nwpu.edu.cn (H.W.)

† These authors contributed equally to this work.

* Correspondence: cyh@mail.xjtu.edu.cn; Tel: +86-029-82665178

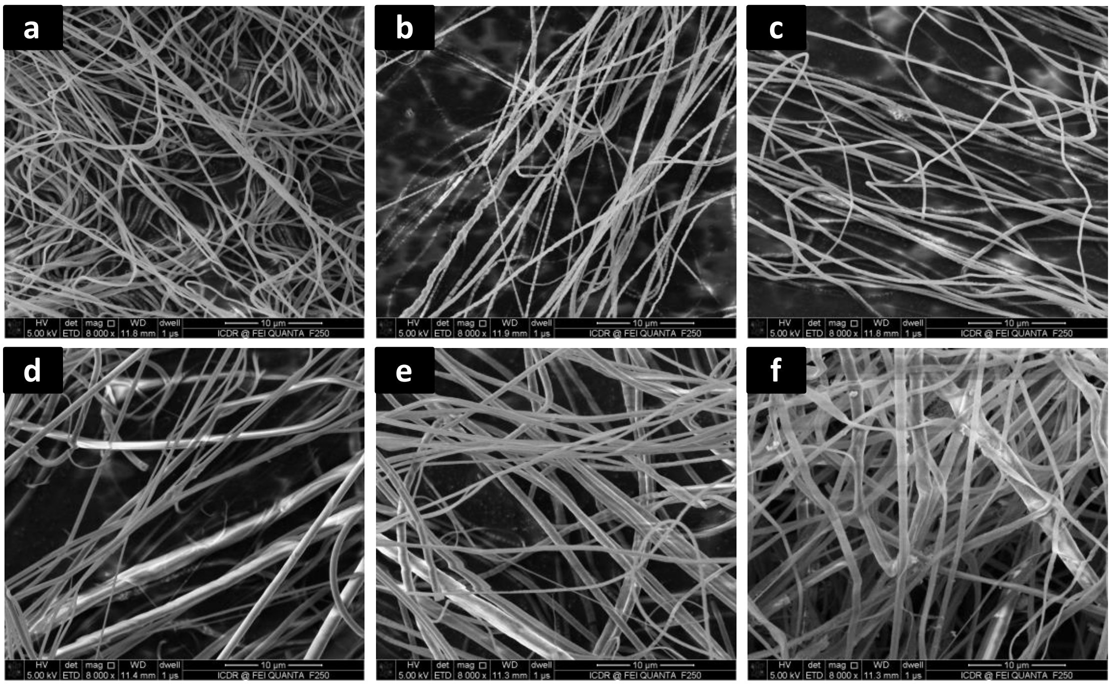

Figure S1. The proportion of PVB/B_2O_3 is 700 mg/350 mg, the voltage is 16.14 kV, the flow is (a)0.75 mL/h (b)1.5 mL/h (c)2.0 mL/h (d)2.5 mL/h (e)4.0 mL/h (f)1.0 mL/h.

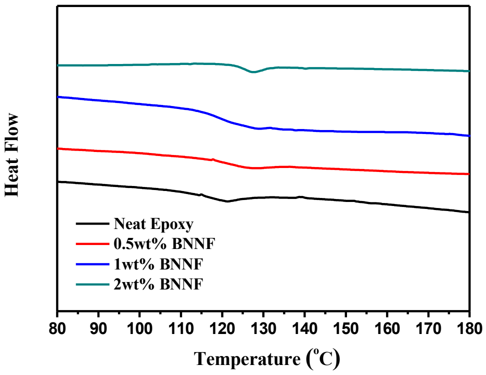

Figure S2. DSC curves of the BNNF/epoxy composites with different filler content under nitrogen atmosphere.

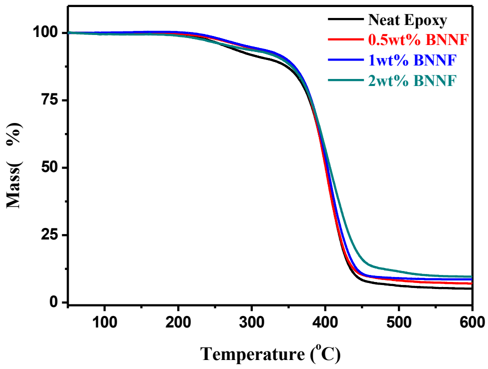

Figure S3. TGA curves of the BNNF/epoxy composites with different filler content under nitrogen atmosphere.

Figure S3 shows the TGA curves of pure epoxy, 0.5 wt%, 1 wt% and 2 wt% BNNF film in nitrogen atmosphere. The TGA curves of all the samples exhibit similar significant weight loss with the temperature range from 300–400 °C, which indicates the mechanism of weight loss of the composites is mainly similar to the pure epoxy. From the Figure, the characteristic thermal decomposition temperature of BNNF/epoxy nanocomposites is higher than that of pure epoxy resin. The thermal conductivity property of the nanofibers of hexagonal boron nitride (h-BN) is much better than that of pure epoxy resin. When the composite is heated, the thermal conductive path of BNNF will transfer the heat effectively and decreases the accumulated energy from heat effect inside the composite, which makes for raising the thermal decomposition temperature of the composites. In addition, if the doping BNNF disperses in the matrix and forms a thermal conductive network, it would be more beneficial to the heat diffusion. To compare the characteristic temperature of these samples, it is found that the characteristic temperature of BNNF/epoxy composites is higher than the pure epoxy resin's but the degree of improvement doesn't increase with the mass fraction of BNNF that the sample of best thermal stability among them is 1 wt% BNNF/EP film.

Table S1. A summary of the detailed data information on thermal conductivity.

Sample	λ (w/m·k)	Deviation (w/ m·k)	α (mm2/s)	Deviation (mm2/s)	C_p (J/g·k)	ρ (g/cm3)
Pure Epoxy	0.162	0.005	0.106	0.003	1.29	1.192
0.5 wt%	0.17	0.010	0.112	0.006	1.26	1.196
1 wt%	0.185	0.004	0.127	0.003	1.21	1.201
2 wt%	0.205	0.011	0.138	0.007	1.22	1.212
